# Supplementary material for: Pyruvate Dehydrogenase Kinase 1 inhibition mediated oxidative phosphorylation enhancement in cartilage promotes osteoarthritis progression
Source: BMC Musculoskelet Disord. 2023 Jul 20;24:597. doi: 10.1186/s12891-023-06585-6 (PMC10357736; doi:10.1186/s12891-023-06585-6)
Supplement: Supplementary file 1 — Additional file 1. [file 12891_2023_6585_MOESM1_ESM.zip › BP.Gsea.1653623667859/GOBP_RNA_STABILIZATION.html]

Details for gene set GOBP\_RNA\_STABILIZATION[GSEA]

|  || Dataset | 55457\_collapsed\_to\_symbols.cls.cls #OA\_versus\_normal.cls.cls #OA\_versus\_normal\_repos |
| Phenotype | cls.cls#OA\_versus\_normal\_repos |
| Upregulated in class | normal |
| GeneSet | GOBP\_RNA\_STABILIZATION |
| Enrichment Score (ES) | -0.521289 |
| Normalized Enrichment Score (NES) | -1.9650769 |
| Nominal p-value | 0.0 |
| FDR q-value | 0.036991827 |
| FWER p-Value | 0.415 |
Table: GSEA Results Summary

  

Fig 1: Enrichment plot: GOBP\_RNA\_STABILIZATION      
 Profile of the Running ES Score & Positions of GeneSet Members on the Rank Ordered List

  

| SYMBOL | TITLE | RANK IN GENE LIST | RANK METRIC SCORE | RUNNING ES | CORE ENRICHMENT || 1 | TRAF3IP2 | TRAF3 interacting protein 2 [Source:HGNC Symbol;Acc:HGNC:1343] | 1939 | 0.368 | -0.1274 | No |
| 2 | MAPK14 | mitogen-activated protein kinase 14 [Source:HGNC Symbol;Acc:HGNC:6876] | 2352 | 0.320 | -0.1391 | No |
| 3 | ANGEL2 | angel homolog 2 [Source:HGNC Symbol;Acc:HGNC:30534] | 2608 | 0.290 | -0.1405 | No |
| 4 | APOBEC1 | apolipoprotein B mRNA editing enzyme catalytic subunit 1 [Source:HGNC Symbol;Acc:HGNC:604] | 2695 | 0.280 | -0.1294 | No |
| 5 | PARN | poly(A)-specific ribonuclease [Source:HGNC Symbol;Acc:HGNC:8609] | 3100 | 0.240 | -0.1455 | No |
| 6 | TENT5A | terminal nucleotidyltransferase 5A [Source:HGNC Symbol;Acc:HGNC:18345] | 3204 | 0.230 | -0.1389 | No |
| 7 | CIRBP | cold inducible RNA binding protein [Source:HGNC Symbol;Acc:HGNC:1982] | 3441 | 0.207 | -0.1440 | No |
| 8 | TENT5C | terminal nucleotidyltransferase 5C [Source:HGNC Symbol;Acc:HGNC:24712] | 3646 | 0.190 | -0.1478 | No |
| 9 | HNRNPD | heterogeneous nuclear ribonucleoprotein D [Source:HGNC Symbol;Acc:HGNC:5036] | 4830 | 0.085 | -0.2344 | No |
| 10 | PAIP1 | poly(A) binding protein interacting protein 1 [Source:HGNC Symbol;Acc:HGNC:16945] | 4851 | 0.083 | -0.2307 | No |
| 11 | DAZ1 | deleted in azoospermia 1 [Source:HGNC Symbol;Acc:HGNC:2682] | 4920 | 0.078 | -0.2310 | No |
| 12 | E2F1 | E2F transcription factor 1 [Source:HGNC Symbol;Acc:HGNC:3113] | 5137 | 0.061 | -0.2439 | No |
| 13 | EIF4ENIF1 | eukaryotic translation initiation factor 4E nuclear import factor 1 [Source:HGNC Symbol;Acc:HGNC:16687] | 5330 | 0.045 | -0.2559 | No |
| 14 | HNRNPA0 | heterogeneous nuclear ribonucleoprotein A0 [Source:HGNC Symbol;Acc:HGNC:5030] | 5485 | 0.033 | -0.2658 | No |
| 15 | TRDMT1 | tRNA aspartic acid methyltransferase 1 [Source:HGNC Symbol;Acc:HGNC:2977] | 6204 | -0.021 | -0.3203 | No |
| 16 | DAZL | deleted in azoospermia like [Source:HGNC Symbol;Acc:HGNC:2685] | 6221 | -0.022 | -0.3201 | No |
| 17 | MYD88 | MYD88 innate immune signal transduction adaptor [Source:HGNC Symbol;Acc:HGNC:7562] | 6638 | -0.052 | -0.3492 | No |
| 18 | RBM10 | RNA binding motif protein 10 [Source:HGNC Symbol;Acc:HGNC:9896] | 6647 | -0.053 | -0.3464 | No |
| 19 | SLC11A1 | solute carrier family 11 member 1 [Source:HGNC Symbol;Acc:HGNC:10907] | 7153 | -0.092 | -0.3798 | No |
| 20 | ELAVL1 | ELAV like RNA binding protein 1 [Source:HGNC Symbol;Acc:HGNC:3312] | 7426 | -0.113 | -0.3938 | No |
| 21 | HNRNPC | heterogeneous nuclear ribonucleoprotein C [Source:HGNC Symbol;Acc:HGNC:5035] | 7437 | -0.114 | -0.3874 | No |
| 22 | TARDBP | TAR DNA binding protein [Source:HGNC Symbol;Acc:HGNC:11571] | 7864 | -0.146 | -0.4112 | No |
| 23 | TRAF2 | TNF receptor associated factor 2 [Source:HGNC Symbol;Acc:HGNC:12032] | 8322 | -0.180 | -0.4353 | No |
| 24 | VIP | vasoactive intestinal peptide [Source:HGNC Symbol;Acc:HGNC:12693] | 9070 | -0.237 | -0.4783 | No |
| 25 | YBX1 | Y-box binding protein 1 [Source:HGNC Symbol;Acc:HGNC:8014] | 9623 | -0.285 | -0.5031 | Yes |
| 26 | IKBKE | inhibitor of nuclear factor kappa B kinase subunit epsilon [Source:HGNC Symbol;Acc:HGNC:14552] | 9646 | -0.287 | -0.4866 | Yes |
| 27 | HNRNPU | heterogeneous nuclear ribonucleoprotein U [Source:HGNC Symbol;Acc:HGNC:5048] | 9847 | -0.306 | -0.4827 | Yes |
| 28 | TENT4A | terminal nucleotidyltransferase 4A [Source:HGNC Symbol;Acc:HGNC:16705] | 10003 | -0.322 | -0.4743 | Yes |
| 29 | TRAF5 | TNF receptor associated factor 5 [Source:HGNC Symbol;Acc:HGNC:12035] | 10085 | -0.329 | -0.4596 | Yes |
| 30 | ELAVL4 | ELAV like RNA binding protein 4 [Source:HGNC Symbol;Acc:HGNC:3315] | 10412 | -0.362 | -0.4619 | Yes |
| 31 | GDNF | glial cell derived neurotrophic factor [Source:HGNC Symbol;Acc:HGNC:4232] | 10475 | -0.370 | -0.4431 | Yes |
| 32 | CSDE1 | cold shock domain containing E1 [Source:HGNC Symbol;Acc:HGNC:29905] | 10559 | -0.379 | -0.4254 | Yes |
| 33 | DKC1 | dyskerin pseudouridine synthase 1 [Source:HGNC Symbol;Acc:HGNC:2890] | 10591 | -0.382 | -0.4035 | Yes |
| 34 | DHX9 | DExH-box helicase 9 [Source:HGNC Symbol;Acc:HGNC:2750] | 10627 | -0.386 | -0.3817 | Yes |
| 35 | LARP1 | "La ribonucleoprotein 1, translational regulator [Source:HGNC Symbol;Acc:HGNC:29531]" | 10964 | -0.425 | -0.3807 | Yes |
| 36 | SYNCRIP | synaptotagmin binding cytoplasmic RNA interacting protein [Source:HGNC Symbol;Acc:HGNC:16918] | 11182 | -0.454 | -0.3687 | Yes |
| 37 | METTL16 | "methyltransferase 16, N6-methyladenosine [Source:HGNC Symbol;Acc:HGNC:28484]" | 11346 | -0.478 | -0.3509 | Yes |
| 38 | NOCT | nocturnin [Source:HGNC Symbol;Acc:HGNC:14254] | 11566 | -0.510 | -0.3355 | Yes |
| 39 | LARP4B | La ribonucleoprotein 4B [Source:HGNC Symbol;Acc:HGNC:28987] | 11695 | -0.531 | -0.3116 | Yes |
| 40 | YBX3 | Y-box binding protein 3 [Source:HGNC Symbol;Acc:HGNC:2428] | 12024 | -0.599 | -0.2990 | Yes |
| 41 | MAPKAPK2 | MAPK activated protein kinase 2 [Source:HGNC Symbol;Acc:HGNC:6887] | 12094 | -0.619 | -0.2650 | Yes |
| 42 | DND1 | DND microRNA-mediated repression inhibitor 1 [Source:HGNC Symbol;Acc:HGNC:23799] | 12266 | -0.667 | -0.2358 | Yes |
| 43 | THRAP3 | thyroid hormone receptor associated protein 3 [Source:HGNC Symbol;Acc:HGNC:22964] | 12426 | -0.717 | -0.2025 | Yes |
| 44 | PABPC1 | poly(A) binding protein cytoplasmic 1 [Source:HGNC Symbol;Acc:HGNC:8554] | 12562 | -0.778 | -0.1635 | Yes |
| 45 | FUS | FUS RNA binding protein [Source:HGNC Symbol;Acc:HGNC:4010] | 12739 | -0.902 | -0.1198 | Yes |
| 46 | TAF15 | TATA-box binding protein associated factor 15 [Source:HGNC Symbol;Acc:HGNC:11547] | 12828 | -1.023 | -0.0615 | Yes |
| 47 | ZFP36 | ZFP36 ring finger protein [Source:HGNC Symbol;Acc:HGNC:12862] | 12846 | -1.052 | 0.0042 | Yes |
Table: GSEA details [plain text format]

  

Fig 2: GOBP\_RNA\_STABILIZATION      
 Blue-Pink O' Gram in the Space of the Analyzed GeneSet

  

Fig 3: GOBP\_RNA\_STABILIZATION: Random ES distribution      
 Gene set null distribution of ES for **GOBP\_RNA\_STABILIZATION**
